# Cannabinoid Receptor 1 Participates in Liver Inflammation by Promoting M1 Macrophage Polarization *via* RhoA/NF-κB p65 and ERK1/2 Pathways, Respectively, in Mouse Liver Fibrogenesis

**DOI:** 10.3389/fimmu.2017.01214

**Published:** 2017-09-28

**Authors:** Lei Tian, Weiyang Li, Le Yang, Na Chang, Xiaoting Fan, Xiaofang Ji, Jieshi Xie, Lin Yang, Liying Li

**Affiliations:** ^1^Department of Cell Biology, Municipal Laboratory for Liver Protection and Regulation of Regeneration, Capital Medical University, Beijing, China

**Keywords:** macrophage polarization, Rho-ROCK signal, siRNA *in vivo*, NF-κB p65, cannabinoid receptor 1

## Abstract

Macrophage M1/M2 polarization mediates tissue damage and inflammatory responses. Cannabinoid receptor (CB) 1 participated in liver fibrogenesis by affecting bone marrow (BM)-derived monocytes/macrophages (BMMs) activation. However, the knowledge of whether CB1 is involved in the polarization of BMMs remains limited. Here, we found M1 gene signatures (including CD86, MIP-1β, tumor necrosis factor, IL-6, and inducible nitric oxide synthase) and the amount of M1 macrophages (CD86^+^ cells, gated by F4/80) were significantly elevated in carbon tetrachloride (CCl_4_)-induced mouse injured livers, while that of M2 type macrophages had little change by RT-qPCR and fluorescence-activated cell sorting (FACS). Our preceding study confirmed CB1 was involved in CCl_4_-induced liver fibrogenesis. Our results noted CB1 expression showed positive correlation with CD86. Blockade of CB1 by its antagonist or siRNA *in vivo* downregulated the mRNA and protein levels of M1 markers using RT-qPCR, western blot, and Cytometric Bead Array (CBA) assays, and reduced the proportion of M1 macrophages. Moreover, chimera mouse models, which received BM transplants from EGFP-transgenic mice or clodronate liposome injection mouse models, in which Kupffer cells were depleted, were performed to clarify the role of CB1 on the polarization of Kupffer cells and BMMs. We found that CB1 was especially involved in BMM polarization toward M1 phenotype but have no effect on that of Kupffer cells. The reason might due to the lower CB1 expression in Kupffer cells than that of BMMs. *In vitro*, we discovered CB1 was involved in the polarization of BMMs toward M1. Furthermore, CB1-induced M1 polarization was apparently impaired by PTX [G(α)_i/o_ protein inhibitor], Y27632 (ROCK inhibitor), and PD98059 [extracellular signal-regulated kinase (ERK) inhibitor], while SB203580 (p38 inhibitor) and compound C (AMPK inhibitor) had no such effect. ACEA (CB1 agonist) activated G(α)_i/o_ coupled CB1, then enlarged GTP-bound Rho and phosphor-ERK1/2, independently. NF-κB p65 nuclear translocation is also a marker of M1 phenotype macrophages. We found that CB1 switched on NF-κB p65 nuclear translocation only depending on G(α)_i/o_/RhoA signaling pathway.

**Conclusion:**

CB1 plays a crucial role in regulating M1 polarization of BMMs in liver injury, depending on two independent signaling pathways: G(α)_i/o_/RhoA/NF-κB p65 and G(α)_i/o_/ERK1/2 pathways.

## Introduction

Macrophage, as an essential component of innate immunity, has emerged as a key role in liver fibrogenesis (1, 2). Activated macrophages are defined as classically activated or M1 type and alternatively activated or M2 type, though this simple assortment does not fully reflect the complex biology of macrophage subsets and the function. M1 type macrophages, characterized by CD86 expression, are stimulated by microbial ligands and cytokines such as lipopolysaccharide and interferon-γ or granulocyte-macrophage colony-stimulating factor and involve in high levels of pro-inflammatory cytokine production, such as MIP-1β, IL-6, inducible nitric oxide synthase (iNOS), and tumor necrosis factor (TNF) (3). Furthermore, a predominance of NF-κB activation promotes M1 macrophage polarization, resulting in cytotoxic and inflammatory functions (3). M1-polarized macrophages mediate tissue damage and initiate inflammatory responses. Uncontrolled M1 macrophages are associated with acute infections, insulin resistance, and obesity-associated inflammation, which may contribute to the development of diseases including cardiovascular, insulin resistance, fatty liver disease, diabetes, atherosclerosis, and cancer (2, 4–6) In contrast, M2 macrophages are characterized by reduced responsiveness to IL-4 and IL-13, resulting in the induction of low levels of proinflammatory mediators and in the upregulation of arginase 1 (Arg1), CD163, and CD206. M2-polarized macrophages play important roles in the protection of the host by decreasing inflammation and promoting tissues repair (7–9). In fact, in many diseases development, there is the M1–M2 switch observed during the transition from acute to chronic stage, which may provide protection against overwhelming uncontrolled inflammation. Therefore, maintaining the balance of M1–M2 type macrophages may be the key point of treating many diseases (4, 10).

Endocannabinoids are lipid mediators that interact with cannabinoid receptors (CBs) to produce effects similar to those of marijuana (11). To date, two types of CBs have been identified: CB1, which is expressed at high levels in the brain but is also present at much lower concentrations yet functionally relevant in peripheral tissues (11), and CB2, which is expressed predominantly in immune and hematopoietic cells (12).

Endocannabinoids, or CB1 and CB2, possess a broad range of biological effects. CB1 activation promotes food intake, increases lipogenesis in adipose tissue and liver, induces insulin resistance and dyslipidemia (13). CB1 also participates in many diseases development, such as hepatocellular carcinoma, diabetes, and renal diseases (14–19). It has been demonstrated that during liver injury, the expressions of endocannabinoids and receptors are increased (20, 21). Our preceding study also showed that biosynthetic enzymes of endocannabinoids are elevated in human fibrotic livers, and the endocannabinoids shows obvious changes in mouse injured liver induced by carbon tetrachloride (CCl_4_) (22).

Although CB1 expression is much lower in immune system, it has a significant role in regulating immune system. CB1 participates in apoptosis of dendritic cell and mediates NK cell function in mice (23). Furthermore, CB1 activation reduces the amount of leukemia inhibitory factor released by peripheral T lymphocytes and mediates IL-2 expression in human peripheral blood T-cells (24). Moreover, it has been reported that CB1 is involved in macrophage activation and function (25–27). Our previous research has shown that CB1 mediates the infiltration and activation of bone marrow-derived monocytes/macrophages (BMMs) in CCl_4_-induced liver injury. Furthermore, the blockade of CB1 suppresses BMM infiltration and ameliorates the inflammation and fibrosis during liver injury (22). However, whether CB1 could mediate BMM polarization in liver injury is still unknown.

In this study, we investigated the effects of CB1 on BMM polarization during liver fibrogenesis. We found that M1 gene signatures were significantly elevated in CCl_4_-induced liver fibrogenesis. Furthermore, the blockade of CB1 reduced the amount of M1 type of BMMs, but had no effect on that of Kupffer cells *in vivo*. Focusing on BMMs, we discovered that CB1 was involved in the polarization of BMMs toward M1 phenotype, depending on two independent signaling pathways: G(α)_i/o_/RhoA/NF-κB p65 and G(α)_i/o_/ERK1/2 pathways.

## Materials and Methods

### Materials

RPMI Medium 1640 was from GIBCO/Invitrogen (Grand Island, NY, USA). FBS was from Hyclone/Thermo Scientific (VIC, Australia). PCR reagents were from Applied Biosystems (Foster City, CA, USA). ACEA (special CB1 agonists), AM281 (CB1 antagonist), AM630 (CB2 antagonist), SB203580 (p38 inhibitor), PD98059 [extracellular signal-regulated kinase (ERK) inhibitor], and compound C [adenosine monophosphate-activated protein kinase (AMPK) inhibitor] were from TOCRIS/R&D (Minneapolis, MN, USA). Y27632 (the inhibitor of Rho-associated protein kinase ROCK), pertussis toxin (PTX), and other common reagents were from sigma (St. Louis, MO, USA). Clodronate liposome (CL) was from YeaSen (Shanghai, China).

### BMM Acquisition

ICR mice aged 3 weeks were sacrificed by cervical dislocation at the time of BM harvest. BM cells were extracted from the tibias and femurs by flushing with culture medium using a 25 G needle. The cells were then passed through a 70-µm nylon mesh (BD Bioscience) and washed three times with PBS. Extracted BM cells were implanted with 2.4 × 10^7^/100 mm culture dish (BD Falcon) and cultured for 7 days in the presence of L929-conditioned medium (replacing culture medium at the third, fifth day). All animal work was performed under the ethical guidelines of the Ethics Committee of Capital Medical University. After 7 days, IL-4 (5 ng/mL) was used to homogenization of BMMs for 5 h.

### BM Transplantation

ICR male mice aged 6 weeks received lethal irradiation (8 Grays) and immediately received transplantation by a tail vein injection of 1.5 × 10^7^ whole BM cells obtained from 3-week-old EGFP transgenic mice. Four weeks later, mice of BM-rebuild were subjected to CCl_4_-induced liver injury. After another 4 weeks, mice were sacrificed and liver tissues were harvested.

### Mouse Models of Liver Fibrosis

A CCl_4_ (1 μL/g BW)/olive oil (OO) mixture (1:9 v/v) was injected into abdominal cavity of mice twice per week. Mice were sacrificed at 1, 3 days and 1, 2, 4 weeks. The liver tissues were harvested. The intraperitoneal injection of AM281 (2.5 mg/kg BW) was performed at 4 h before CCl_4_ administration (*n* = 6). The CLs (from Vrije University Amsterdam) were injected 24 h before CCl_4_ administration *via* caudal vein. All animal work was conformed to the Ethics Committee of Capital Medical University and in accordance with the approved guidelines (specific institutional approval number of animal experiment: AEEI-2014-131).

### Fluorescence-Activated Cell Sorting (FACS)

Isolation of mouse liver macrophages was as described in this section. Subsequently, antibodies: PE-CD86 (BD Biosciences, Franklin Lakes, NJ, USA), and its isotype-matched negative control antibodies were added to the cell suspension. After 15 min of incubation in the dark, the cells were washed with PBS and subjected to FACS. FACS was performed on a FACSAria and analyzed with FACSDiva4.1 (BD Biosciences).

### RNA Interference (RNAi) *In Vivo*

Mouse chemically modified and stable siRNAs of CB1 and Invivofectamine 3.0 reagent were from Invitrogen (Thermo Scientific, PA, USA). The method is briefly described as follows. The solution of siRNA duplex (1.2 mg/mL) 50 µL was mixed with 50 µL Buffer. The solution was mixed with 100 µL Invivofectamine 3.0 reagent, then incubated at 50°C for 30 min. The complex was diluted with 1 mL PBS. The solution (10 µL/g BW) was injected into mouse tail vein at 4 h before the first time of CCl_4_ administration (*n* = 6).

### Western Blot Analysis

Western blot analysis was performed with 50 µg of protein extract. Antibodies were as follows: rabbit anti-iNOS polyclonal antibody (1:200, St. Louis, MO, USA), rabbit anti-NF-κB p65 polyclonal antibody (1:1,000), rabbit anti-ERK1/2 polyclonal antibody (1:1,000), and rabbit anti-phorspho-ERK1/2 polyclonal antibody (1:1,000) (Cell Signaling, Beverly, MA, USA); rabbit anti-β-tubulin and anti-GAPDH monoclonal antibodies (1:1,000, Abcam, UK). ODYSSEY goat anti-rabbit IRDye^®^ 800 CW antibody (1:10,000, LI-COR, NE, USA) was used as secondary antibody. The bands were displayed using ODYSSEY and quantified by Odyssey v3.0 software. β-tubulin or GAPDH was as reference.

### Immunofluorescence Staining

Bone marrow-derived monocyte/macrophage that had been cultivated for 7 days were fixed by 4% paraformaldehyde for 30 min and penetrated by 0.5% Triton X-100 (Amresco, OH, USA) for 15 min. After blocked with 3% BSA (Roche, Switzerland), they were incubated with anti-NF-κB p65 rabbit polyclonal antibodies (1:40; Cell Signaling, Beverly, MA, USA) or anti-phorspho-ERK1/2 (1:40; Cell Signaling, Beverly, MA, USA). FITC-conjugate affinipure goat-anti-rabbit IgG (1:100, Jackson Immunoresearch, PA, USA) was as secondary antibody. Nuclei were stained with DAPI. For high content analysis, the plates were imaged on Thermo Scientific CellInsight personal cell imaging platform (Cellomics, Inc., Thermo Fisher Scientific Inc., Waltham, MA, USA). 48 fields were automatically acquired by the software, corresponding to at least 4,000 cells. Cytoplasmic or nuclear fluorescence intensity of each well was analyzed by Cellomics Cell Health Profiling BioApplication software.

The liver specimen was fixed in 4% paraformaldehyde and frozen sections of 6 mm were used for immunofluorescence and paraffin sections for H&E. Frozen sections were incubated with F4/80 antibody as the first antibody and Cy3-conjugated goat anti-rat IgG (1:100, Jackson ImmunoResearch Laboratories) as the secondary antibody. Finally, the sections were stained with DAPI and observed under a confocal microscope (LSM510, Carl Zeiss MicroImaging, Jena, Germany).

### RT-qPCR

Total RNA was extracted from liver frozen specimens or cultured BMMs with or without treatments using an RNeasy kit (Qiagen, Hilden, Germany). RT-qPCR was performed in an ABI Prism 7300 sequence detecting system (Applied Biosystems, Foster City, CA, USA). Primers sequences are listed in Table 1.

**Table 1:** Primer sequence.

Mouse		Sequence
18s rRNA	Sense	GTAACCCGTTGAACCCCATT
	Antisense	CCATCCAATCGGTAGTAGCG
IL-6	Sense	CTCTGGGAAATCGTGGAAATG
	Antisense	AAGTGCATCATCGTTGTTCATACA
Tumor necrosis factor	Sense	GGCAGGTTCTGTCCCTTTCA
	Antisense	CTGTGCTCATGGTGTCTTTTCTG
Inducible nitric oxide synthase	Sense	TGACGGCAAACATGACTTCAG
	Antisense	GGTGCCATCGGGCATCT
MIP-1β	Sense	CCAGCTCTGTGCAAACCTAACC
	Antisense	GCCACGAGCAAGAGGAGAGA
CD86	Sense	TCCAAGTTTTTGGGCAATGTC
	Antisense	CCTATGAGTGTGCACTGAGTTAAACA
Arginase1	Sense	GTCTGGCAGTTGGAAGCATCT
	Antisense	GCATCCACCCAAATGACACA
CD163	Sense	CAGGTGTTATCTGCTCCGAGTTC
	Antisense	CCCCATGTACCATTGTAAAACACTT
CD206	Sense	GGTGGAAGAAGAAGTAGCCT
	Antisense	GAAGGGTCAGTCTGTGTTTG
CB1	Sense	GGCGGTGGCCGATCTC
	Antisense	CGGTAACCCCACCCAGTTT

### Measurement of Cytokines and Chemokines by Cytometric Bead Array (CBA)

Applied BD CBA Mouse Flex Kit (Catalog No.558266), briefly as follows: liver tissues (~40 mg) or BMM (a density of 7,000 cells/cm^2^) supernatant homogenized into 40 µL and mixed in 40 µL buffer with IL-6 (Catalog No. 5580301), TNF (Catalog No. 558299) and MIP-1β (Catalog No. 558449). The sample was acquired on a FACSAria and analyzed with FACSDiva4.1 (BD Biosciences).

### Mouse Primary Liver Macrophage Isolation

Primary liver macrophages were isolated from treated adult mice. First, anesthetized and heparinized mice were subjected to a midline laparotomy and cannulation of the portal vein followed by liver perfusion with an EGTA-chelating perfusion buffer (EGTA: 190 mg, glucose: 900 mg, HEPES: 10 mL of 1 M stock solution, KCl: 400 mg, Na_2_HPO_4_–12H_2_O: 305 mg, NaCl: 8 g, NaH_2_PO_4_–2H_2_O: 88 mg, and NaHCO_3_: 350 mg, made up to 1 L with dH_2_O). After perfusion with 0.4% collagenase buffer (CaCl_2_–2H_2_O: 560 mg, HEPES: 10 mL of 1 M stock solution, KCl: 400 mg, Na_2_HPO_4_–12H_2_O: 305 mg, NaCl: 8 g, NaH_2_PO_4_–2H_2_O: 88 mg, NaHCO_3_: 350 mg, and collagenase IV: 400 mg, made up to 1 L with dH_2_O), livers were teared and cells dispersed in saline; non-parenchymal cells were separated using low-speed centrifugation and 40% percoll density gradient centrifugation. F4/80 was used to sorted macrophages using FACS. Isolated macrophages were used to perform qPCR and FACS.

### RNAi *In Vitro*

The ON-TARGETplus mouse CB1 siRNA smart pool (L-042461-00), CB2 siRNA smart pool (L-062503-00), and non-targeting control pool (D-001810-10-05) were from Dharmacon (Thermo Scientific, PA, USA). 40–50% confluent BMMs were prepared in 60-mm dishes. Transient transfection of siRNA (40 nmol/L) was performed by using Lipofectamine RNAiMAX (Invitrogen, Carlsbad, CA, USA) as recommended by the manufacturer. Control cells were treated with 40 nmol/L RNA interference negative control duplexes (scramble siRNA). After 48 h, cells were used to perform the following assay.

### Measurement of Activity of Small GTPases by Pull-Down Assay

Bone marrow-derived monocyte/macrophage with or without AM281, PTX, or PD98059 pretreatment were exposed to ACEA for different times, then BMM was lysed in lysis buffer, active RhoA was extracted using pull-down and detection kit (Catalog No. 16116, Thermo Scientific Pierce Biotechnology, IL, USA), finally, active and total Rho was showed by Western blot.

### Statistical Analysis

Results are expressed as mean ± SEM. Statistical significance was determined by Student’s *t*-test or ANOVA. Comparisons between two independent groups were performed using a two-sample *t*-test. Comparisons between multiple groups were performed by one-way analysis of variance with *post hoc* Tukey’s multiple comparison tests. Correlation coefficients were calculated by Pearson’s test. *P* < 0.05 was considered significant.

## Results

### M1 and M2 Gene Signatures in Livers Show Changes during Liver Fibrogenesis

**Figure 1:**
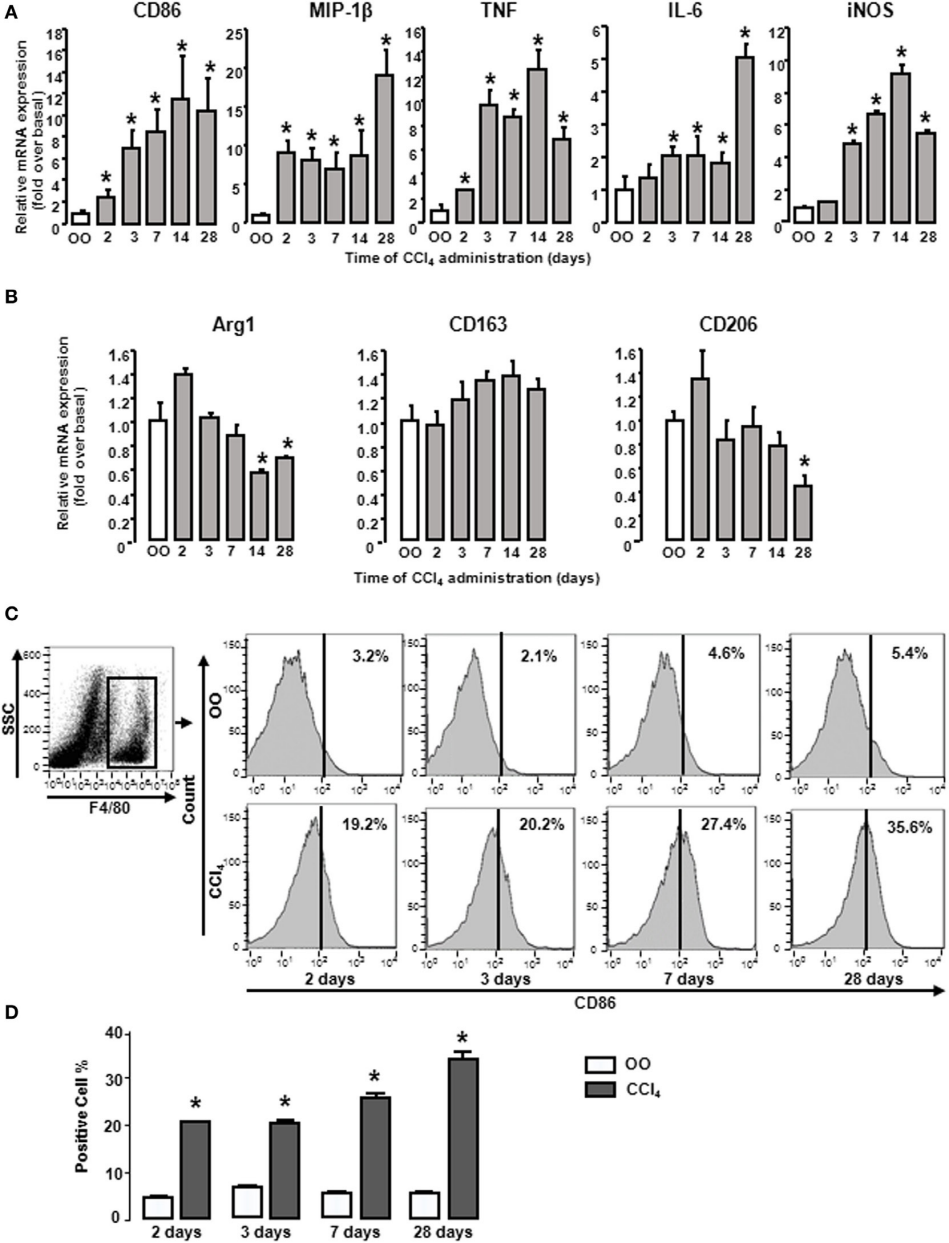
M1 and M2 gene signatures in mouse livers showed changes during CCl_4_ administration. Expressions of M1 and M2 markers were determined by RT-qPCR and fluorescence-activated cell sorting (FACS). At different times after CCl_4_ administration, mRNA levels of M1 markers (CD86, MIP-1β, tumor necrosis factor, IL-6, and inducible nitric oxide synthase) **(A)** and M2 markers (Arg1, CD163, and CD206) **(B)** were measured. Primary liver macrophages (F4/80^+^) sorted from treated livers. Representative flow cytometric histograms of total M1-type macrophages **(C)**. Quantification of FACS for cell proportion **(D)**. *P < 0.05 compared with the group of olive oil administration on the same time (*n* = 6/group).

During liver fibrogenesis, the amount of macrophages was significantly increased in livers (28), yet their characters of polarization or the dynamic changes of M1 and M2 types are unclear. Thus we first detected the dynamic changes of M1 and M2 gene signatures in mouse livers at different times after CCl_4_ treatment. CD86 (M1 type macrophage marker) mRNA was significantly upregulated from 2 days of CCl_4_ administration and reached the peak at 2 weeks. After that, CD86 mRNA sustained around 10-fold of the initial level. Furthermore, we also investigated other M1 gene signatures, including MIP-1β, TNF, IL-6, and iNOS in injured livers. The mRNA level of MIP-1β rose rapidly and reached the peak at 4 weeks. iNOS and TNF mRNA also increased and got to the topmost level after 2 weeks, whereas IL-6 mRNA was slightly increased with a maximal increase after 4 weeks (Figure 1A). Considering M2 gene signatures, Arg1 mRNA had no changes at the early injured period and generated a slight decrease during chronic stage. CD206 mRNA had little changes at early injured stage and generated a violent decline at 4 weeks. CD163 expressions had no changes (Figure 1B). Next, we detected the profile of M1 type macrophage amount. Liver macrophages were isolated from livers with different treatments by F4/80^+^ gating (Figure 1C left). FACS analysis showed that the proportion of total M1 type macrophages in CCl_4_-induced damaged livers was upregulated markedly from 2 days of CCl_4_ administration compared with that in OO-treated liver and reached the peak at 4 weeks (Figures 1C,D). These results indicated M1-, but not M2, type macrophages might play important roles in liver injury.

### CB1 Positively Correlated with Macrophage M1-Type Markers (CD86, etc.) and Blockade of CB1 Especially Reduced BMM Polarization toward M1 Phenotype in Injured Liver

**Figure 2:**
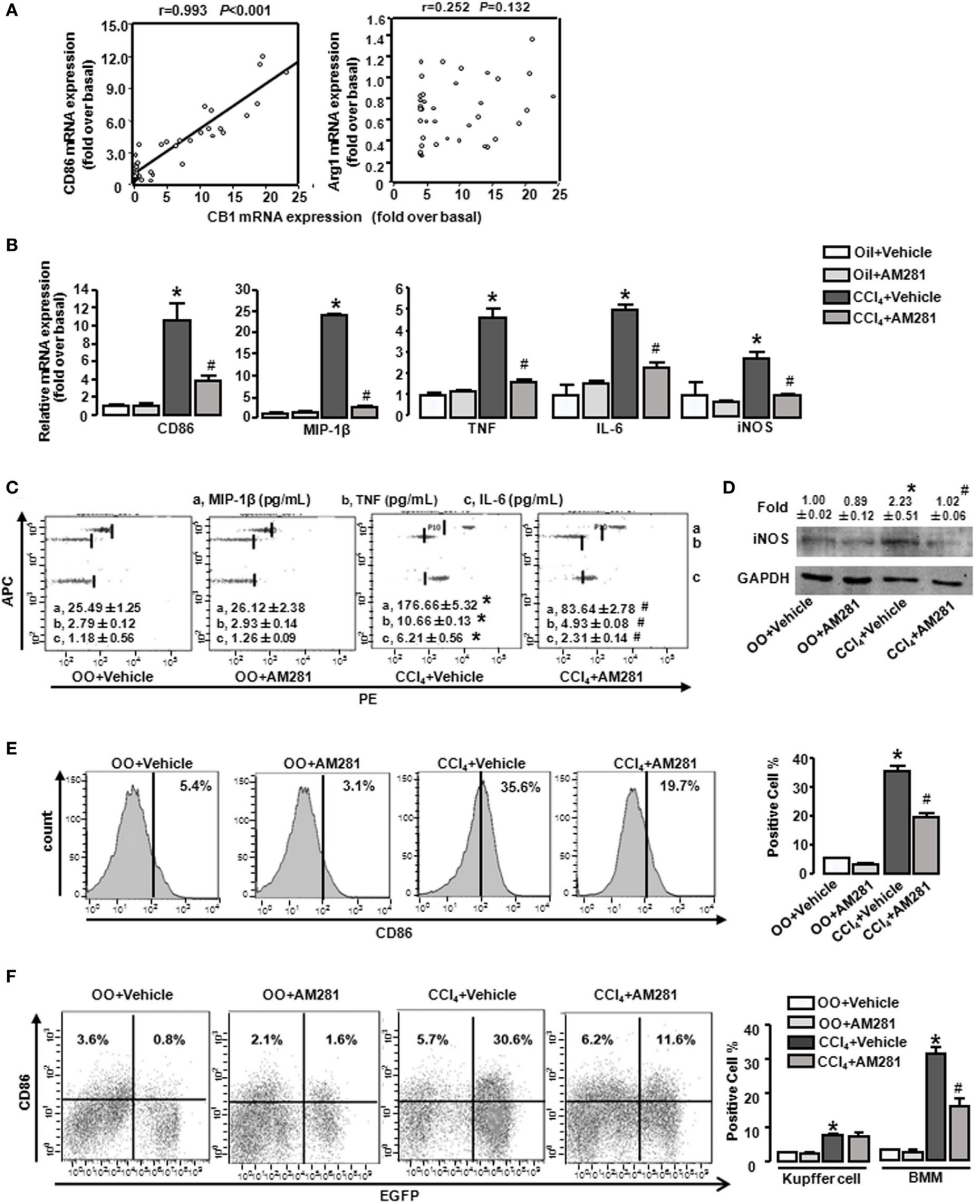
CB1 positively correlated with M1-type markers (CD86) and blockade of CB1 especially reduced the proportion of M1-type bone marrow-derived monocytes/macrophages (BMMs) in injured liver. The correlation between mRNA levels of CB1 and CD86 and arginase1 **(A)**. M1 markers were measured by RT-qPCR, western blot, and CBA. After CCl_4_ administration for 4 weeks, the mRNA levels of M1 type markers were measured **(B)**. Representative scatter plots of CBA are shown **(C)**. Three bead populations represented MIP-1β, TNF, and IL-6 based on allophycocyanin fluorescence intensity from high to low. The number presented the concentration of these proteins. The expression of inducible nitric oxide synthase was measured by western blot **(D)**. Among macrophages of mouse liver that were isolated, total M1-type macrophages (CD86^+^) **(E)**, M1-type cell of BM origin (EGFP^+^CD86^+^) [**(F)** left] and M1 type of Kupffer cell (EGFP^−^CD86^+^) [**(F)** left] were analyzed by fluorescence-activated cell sorting (FACS). Quantification of FACS for cell proportion [**(E)** right and **(F)** right]. *P < 0.05 compared with olive oil plus vehicle group, ^#^*P* < 0.05 compared with CCl_4_ plus vehicle group (*n* = 6/group).

Our preceding study demonstrated that CB1 was involved in CCl_4_-induced liver injury and CB1 expression was increased (22). Here, we undertook correlation analysis between mRNA expression level of CB1 and CD86 or Arg1. In mouse CCl_4_-injured livers, the mRNA levels of CD86 positively correlated with CB1 (*r* = 0.993, *P* < 0.001), whereas the mRNA level of Arg1 showed no correlations with it (Figure 2A). We also noted that CB1 mRNA exerted positive correlations with MIP-1β, TNF, IL-6, or iNOS (Table 2). However, all of M2 gene signature (including Arg1, CD163, and CD206) expressions had no correlations with CB1 (Table 2). Collectively, these results suggested that CB1 might play a crucial role in regulating M1 polarization of macrophages during liver fibrogenesis.

**Table 2:** The correlation between mRNA levels of CB1 and M1-related markers or M2-related markers in livers.

		*CB1*	
		*r*	*P value*
M1-related markers	MIP-1β	0.970	<0.01
	Inducible nitric oxide synthase (iNOS)	0.995	<0.01
	IL-6	0.653	<0.01
	Tumor necrosis factor (TNF)	0.998	<0.01

M2-related markers	CD163	0.081	0.676
	CD206	−0.132	0.465

*The mRNA expression of CB1, M1 markers (MIP-1β, iNOS, IL-6, TNF), and M2 markers (CD163 and CD206) was quantified using RT-qPCR. The relationship between CB1 and M1 markers/M2 markers was analyzed by regression analysis. Correlation coefficients (*r*)*.

To figure out the role of CB1 on macrophage polarization in insulted livers, AM281 (CB1 antagonist) administration was performed in the model. Four weeks of AM281 injection markedly reduced, the mRNA levels of M1 markers in injured livers compared with that in the injured livers without AM281 pretreatment (Figure 2B). Furthermore, the blockade of CB1 restrained the protein levels of MIP-1β, TNF, IL-6 (by CBA) (Figure 2C), and iNOS (by western blot) (Figure 2D) in injured livers. In addition, FACS analysis showed that the amount of total M1-type macrophages (F4/80^+^/CD86^+^) in CCl_4_-induced damaged liver was decreased after AM281 administration (Figure 2E).

It has been reported that there are two kinds of macrophages in damaged livers, the tissue-resident macrophages, Kupffer cells, and the infiltrated macrophages, BMMs. To further clarify the role of CB1 on Kupffer cell and BMM polarization, BM in the irradiated mice were reconstituted by transplantation of the genetic EGFP-labeled BM cells. Then, the BM rebuilt mice were induced liver injury by CCl_4_ administration. After 4 week administration, we isolated liver macrophages (F4/80^+^ cells). EGFP^+^/CD86^+^ (M1 type of BMM) proportion in macrophages of damaged liver was increased after CCl_4_ administration for 4 weeks and the amount of M1 type of Kupffer cell (EGFP^−^/CD86^+^) was also upregulated. But the increase of Kupffer cells was much less than that of BMMs. Specifically, almost 80% of M1-polarized macrophages are BMMs, which implied M1 type of BMMs might act as an important part of liver fibrogenesis. After AM281 pretreatment, the increased amount of EGFP^+^/CD86^+^ was significantly limited, while AM281 pretreatment had no effect on the amount of M1-type Kupffer cells (Figure 2F). Therefore, we thought that CB1 was involved in mediating the proportion of M1 type BMMs.

**Figure 3:**
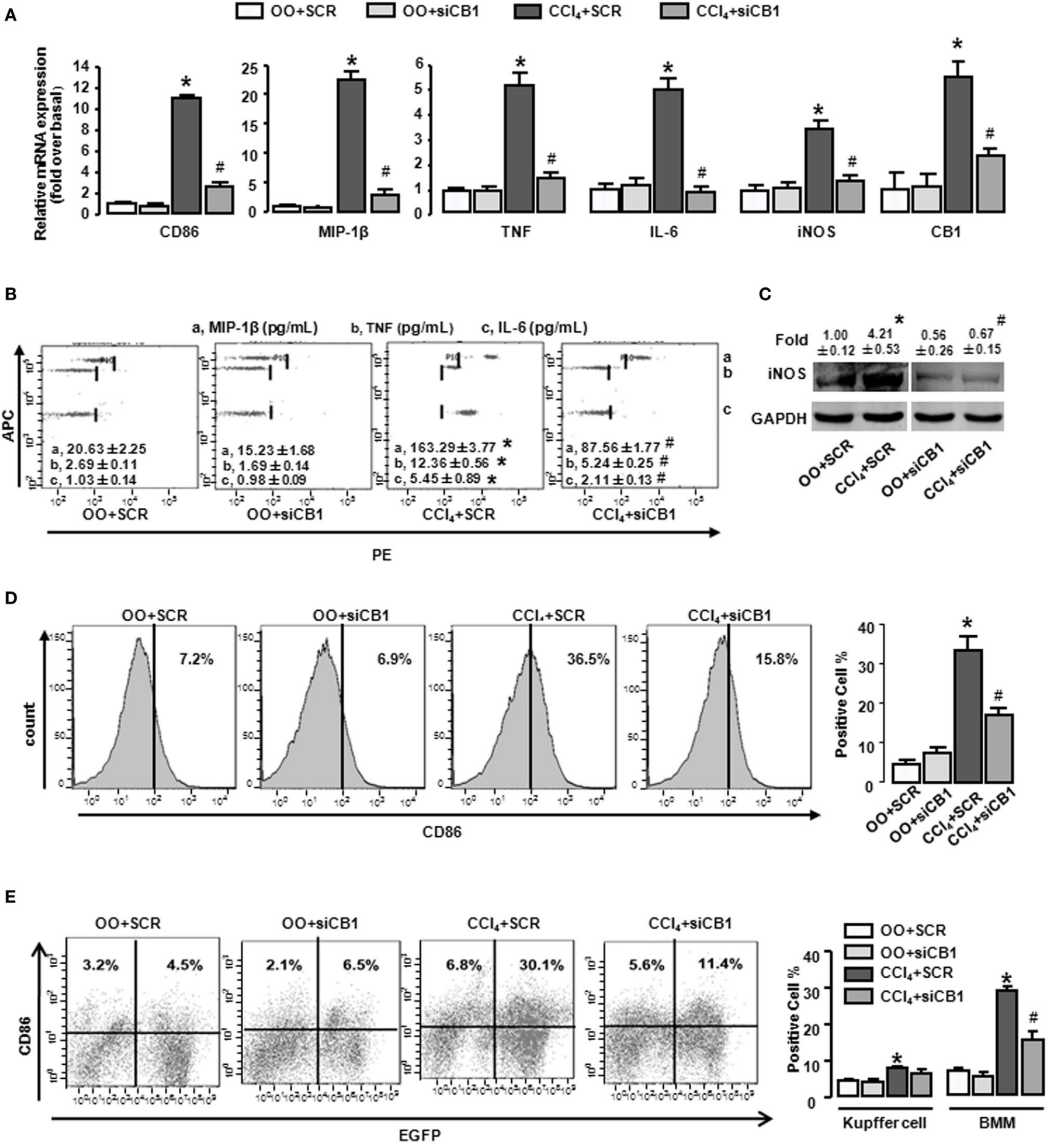
CB1-siRNA *in vivo* reduced the polarization of bone marrow-derived monocytes/macrophages (BMMs) toward M1 phenotype. Effectiveness of CB1-siRNA *in vivo* was measured by RT-qPCR **(A)**. M1 markers were measured by RT-qPCR **(A)**, western blot **(C)**, and CBA **(B)**. Among macrophages of mouse liver that were isolated, total M1-type macrophages **(D)**, M1 type of BM origin [**(E)** left], and M1 type of Kupffer cell [**(E**) left] were analyzed by fluorescence-activated cell sorting (FACS). Quantification of FACS for cell proportion [**(D)** right and **(E)** right]. *P < 0.05 compared with olive oil plus vehicle group, ^#^*P* < 0.05 compared with CCl_4_ plus vehicle group (*n* = 6/group).

In order to confirm our conclusion, we adopted an established model in which chemically modified and stable siRNAs of CB1 was injected rapidly into the mouse tail vein, using a “hydrodynamic transfection method” (29). CB1-siRNA successfully downregulated each mRNA level by 61.3% in liver (Figure 3A), which confirmed the effectiveness of the siRNA. CB1 siRNA pretreatment decreased the mRNA and protein levels of M1 markers in CCl_4_-damaged livers (Figures 3A–C). Moreover, FACS analysis for CD86 showed that the results of CB1 siRNA administration were consisted with AM281 pretreatment results (Figures 3D,E).

### Blockade of CB1 Especially Reduced BMM Polarization toward M1 Phenotype and Have No Effect on That of Kupffer Cells *In Vivo*

**Figure 4:**
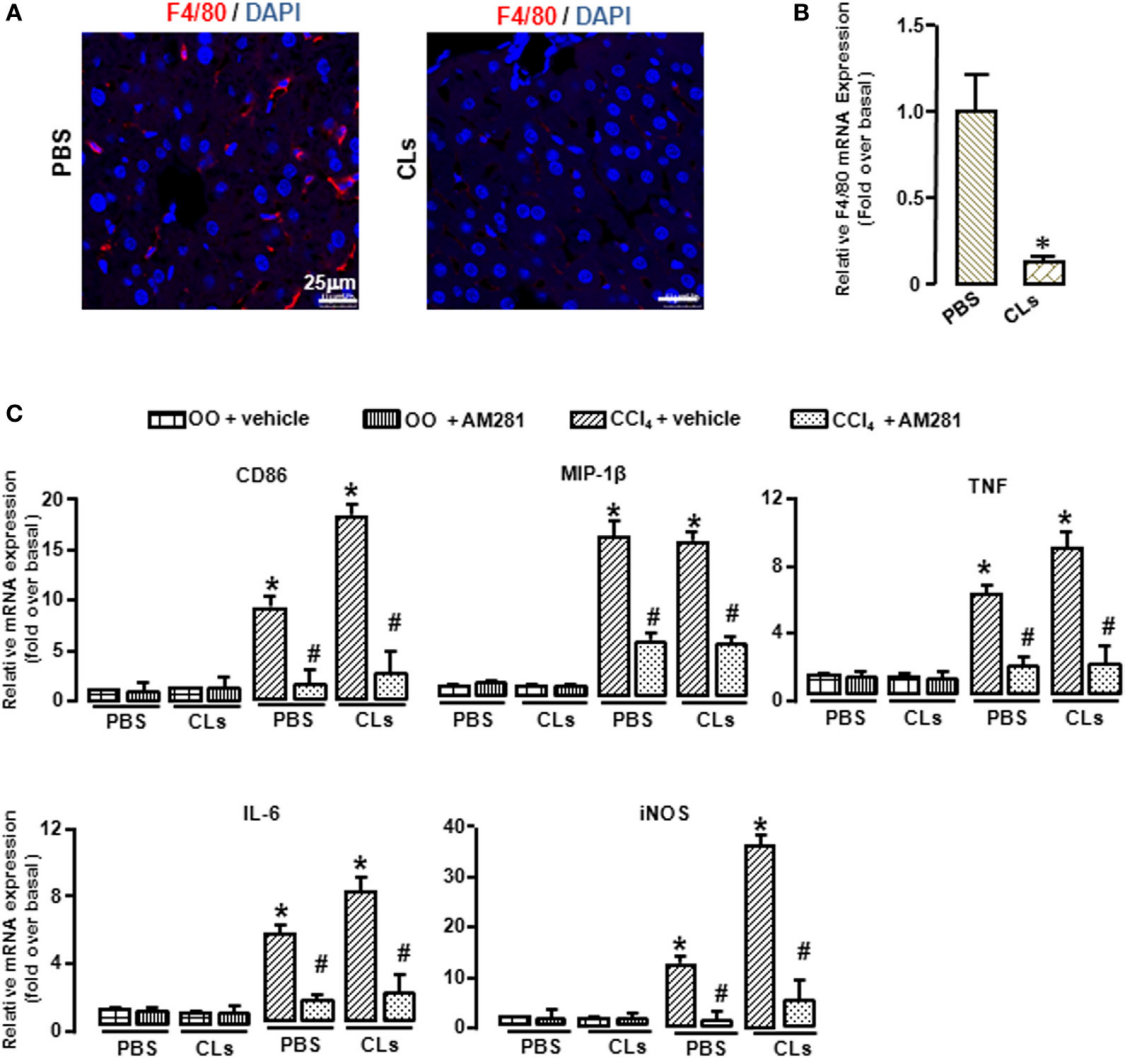
Blockade of CB1 reduced the polarization of bone marrow-derived monocytes/macrophages (BMMs) toward M1 phenotype but had no effect on that of Kupffer cells *in vivo*. The clodronate liposome injection was performed to delete Kupffer cell. Elimination efficiency was measured by immunofluorescence and RT-qPCR. Representative liver images were showed by confocal microscopy to track macrophages (F4/80^+^, red) **(A)**. DAPI was used to visualize nuclei (blue). Scale bars, 25 µm. The mRNA level of F4/80 was measured **(B)**. M1 markers were determined by RT-qPCR **(C)**. *P < 0.05 compared with the group of olive oil administration on the same time (*n* = 6/group).

**Figure 5:**
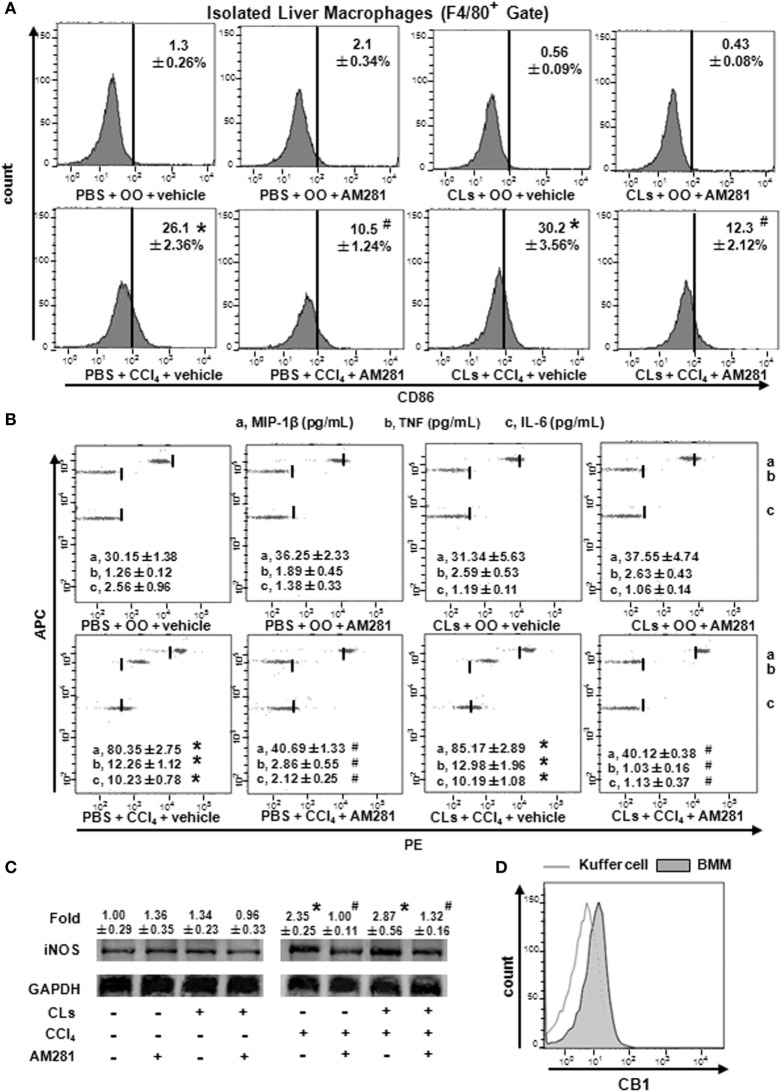
Blockade of CB1 especially reduced bone marrow-derived monocyte/macrophage (BMM) polarization toward M1 phenotype and have no effect on that of Kupffer cells *in vivo*. The clodronate liposome injection was performed to delete Kupffer cell. Among macrophages of mouse liver that were isolated, total M1-type macrophages **(A)** were analyzed by fluorescence-activated cell sorting (FACS). M1 markers were measured by western blot **(C)** and CBA **(B)**. CB1 expression on Kupffer cells and BMMs were detected by FACS **(D)**. *P < 0.05 compared with olive oil plus vehicle group, ^#^*P* < 0.05 compared with CCl_4_ plus vehicle group (*n* = 6/group).

To confirm the role of CB1 on BMMs and Kupffer cells *in vivo*, we deleted Kupffer cells using CLs (30) and performed CCl_4_ administration with or without AM281 pretreatment. Four weeks later, we examined M1 marker expressions in macrophages isolated from injured livers to exclude the role of Kupffer cells. The results of immunofluorescence assay and RT-qPCR showed the elimination efficiency (Figures 4A,B). We found that AM281 pretreatment on reducing mRNA levels of M1 marker were not affected after Kupffer cell deletion (Figure 4C). Furthermore, we used FACS to detect the amount of M1-type BMM in the isolated cell. Our result showed that the decreased amount of CD86^+^ macrophages reduced by AM281 pretreatment was unchanged after CLs injection (Figure 5A). We also measured the protein levels of M1 markers. Consistent with the mRNA results, AM281 pretreatment on reducing protein levels of M1 marker were not affected after Kupffer cell deletion (Figures 5B,C). These results indicated that CB1 was especially involved in BMM polarization but have no effect on that of Kupffer cells. To figure out the reason of CB1 different ability on the two kinds of macrophages, we measured CB1 expression on Kupffer cells and BMM by FACS. We noted that the expression of CB1 on BMM was higher than that of Kupffer cell (Figure 5D). These results confirmed that CB1 was involved in M1 polarization of BMMs in CCl_4_-induced liver fibrogenesis.

### Activation of CB1 Promotes BMM Polarization toward M1 Phenotype *In Vitro*

**Figure 6:**
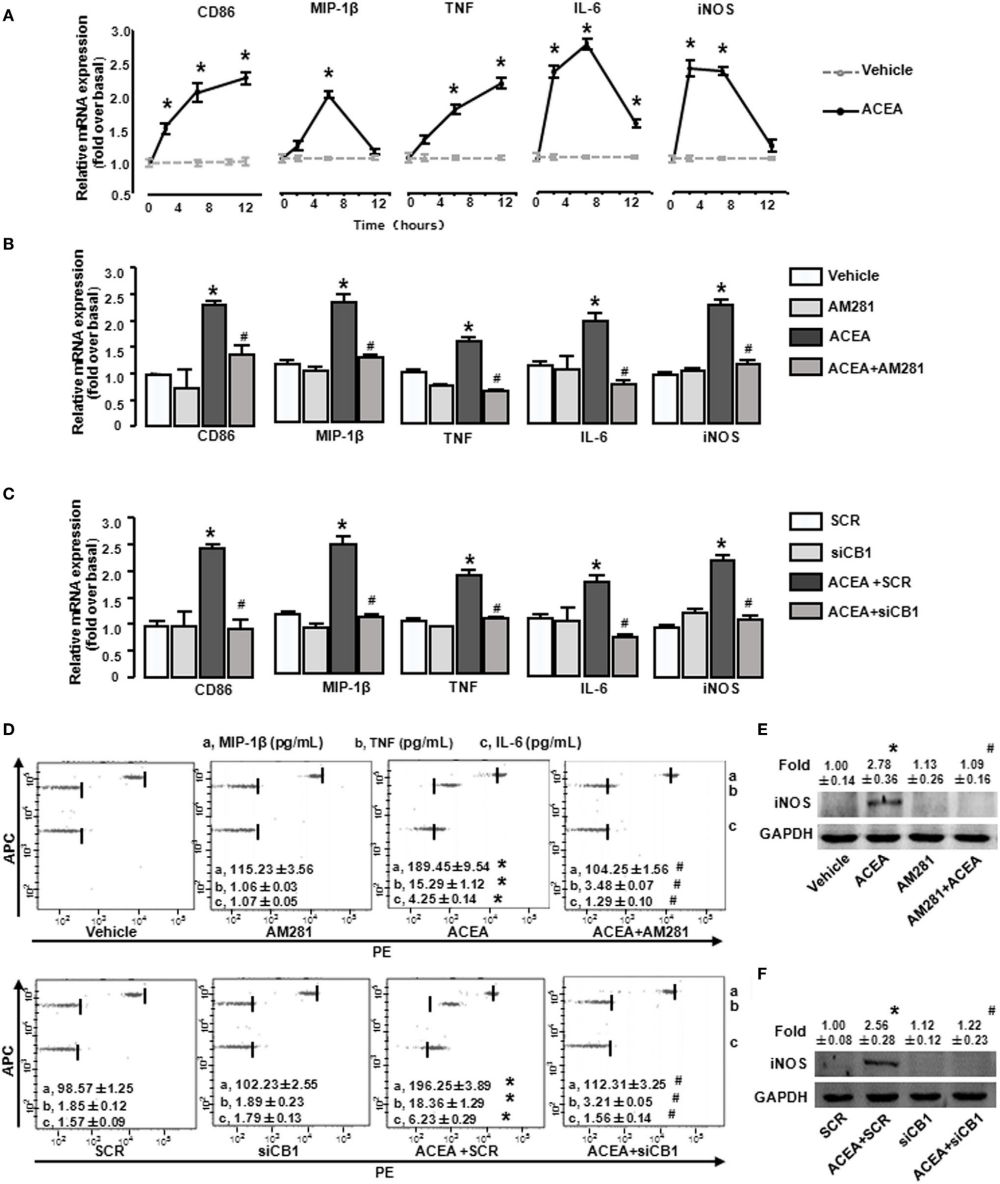
Activation of CB1 promotes M1 marker expressions in bone marrow-derived monocytes/macrophages (BMMs). BMMs were exposed to ACEA for different times. The mRNA levels of M1 markers were measured **(A)**. BMMs pretreated with 10 µmol/L AM281 or CB1 RNA interference was exposed to ACEA for 5 h. M1 markers were measured by RT-qPCR **(B,C)**, western blot **(E,F)**, and CBA **(D)**. *P < 0.05 compared with control, ^#^*P* < 0.05 compared with the same treated group without inhibitors (*n* = 4).

We then investigated the effects of CB1 on BMM polarization *in vitro*. ACEA (1 µmol/L) promoted the mRNA levels of CD86 and TNF at 2, 6, and 12 h, implying that the activation of CB1 effectively promoted M1 polarization. Furthermore, CB1 activation also increased iNOS, MIP-1β, and IL-6 expressions at 2 and 6 h (Figure 6A). When BMMs were pretreated with AM281, ACEA-induced elevations of M1 gene signature mRNA levels were suppressed (Figure 6B). To confirm the critical role of CB1, we employed CB1-specifc siRNA. We first measured that CB1 siRNA effectively silenced the corresponding target gene at protein levels (Figure S1A in Supplementary Material). Silencing CB1 expression with siRNA abrogated the M1 polarization of BMMs induced by ACEA (Figure 6C). Actually, ACEA also showed low CB2 affinity, so we applied CB2 antagonist (AM630) and CB2 siRNA to BMMs. But, we found that AM630 (10 µmol/L) and CB2 knockdown hardly inhibited ACEA-mediated M1 polarization of BMMs (Figures S1B,C in Supplementary Material). Furthermore, CB1 activation also upregulated the protein levels of MIP-1β, IL-6, TNF, and iNOS, especially TNF, which was upregulated over 10-fold. Additionally, the increase protein levels of M1 markers induced by ACEA were abrogated by AM281 or siCB1 (Figures 6D–F). TNF expression was even reverted to the initial phase after AM281 or siCB1 pretreatment. Altogether, these results suggested that CB1 activation exerted a powerful action on BMM polarization toward M1 type.

### CB1-Mediated BMM Polarization toward M1 Depending on G(α)_i/o_, RhoA, ERK1/2, and NF-κB

**Figure 7:**
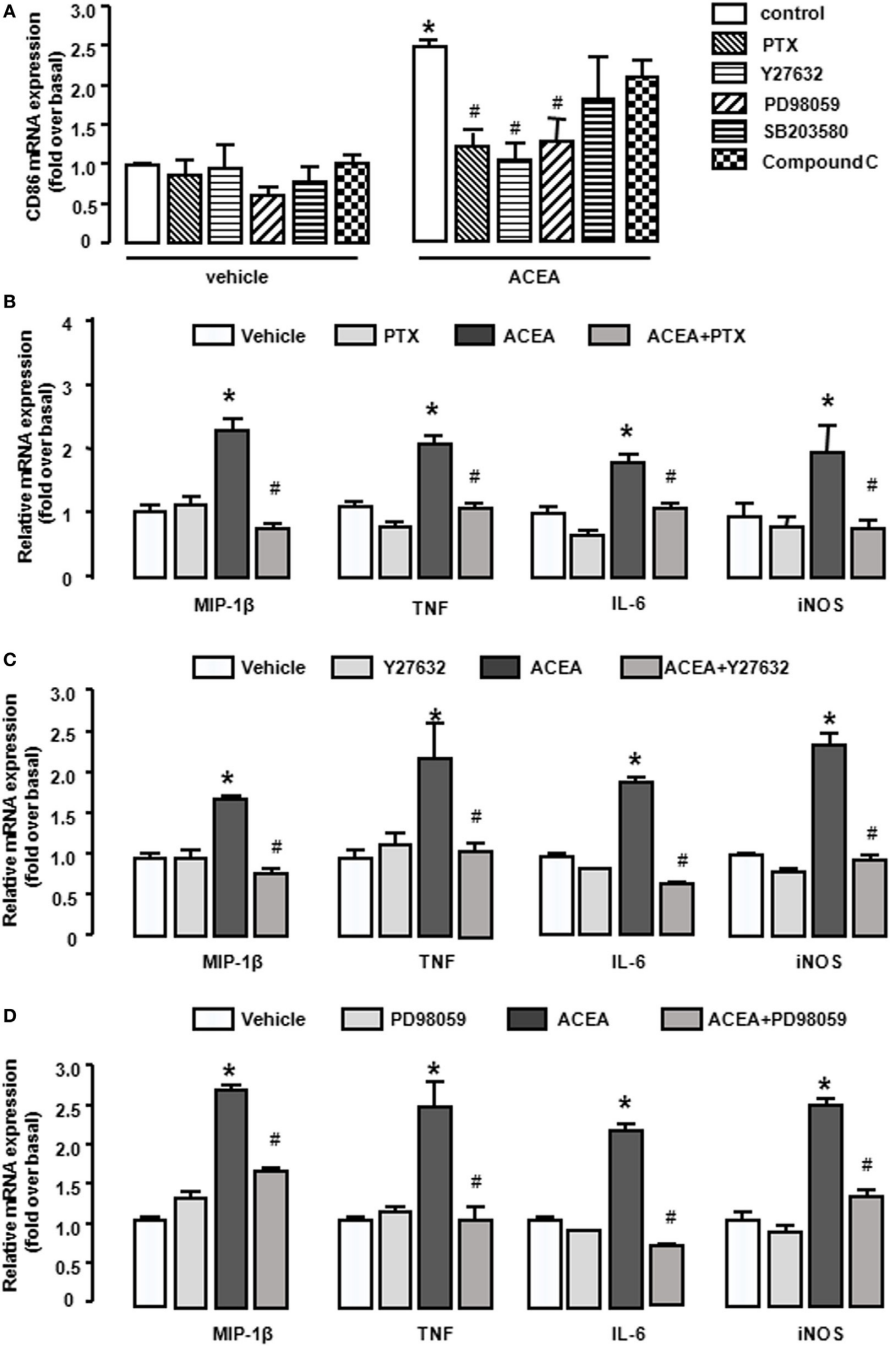
CB1 activation promoted bone marrow-derived monocyte/macrophage (BMM) polarization toward M1 through G(α)_i/o_, RhoA, and ERK1/2. BMMs were pre-treated with pertussis toxin (20 ng/mL), Y27632 (10 µmol/L), SB203580 (10 µmol/L), PD98059 (10 µmol/L), compound C (10 µmol/L) for 1 h, and followed by 1 µmol/L ACEA treatment for another 6 h. CD86 mRNA expression **(A)** and M1 other gene signatures **(B–D)** were evaluated by RT-qPCR. *P < 0.05 compared with control, ^#^*P* < 0.05 compared with the same treated group without inhibitors (*n* = 4).

**Figure 8:**
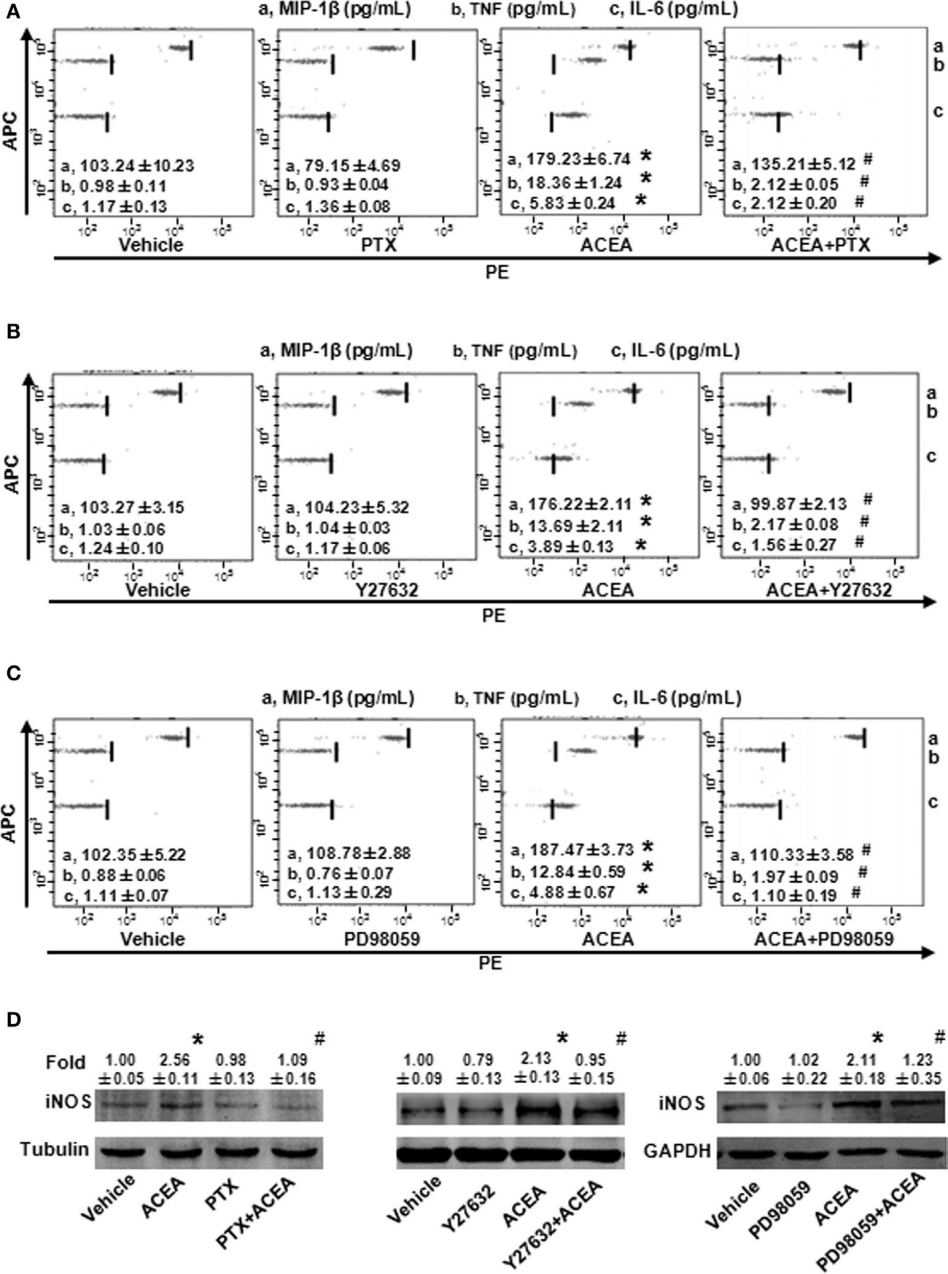
G(α)_i/o_, RhoA, and ERK1/2 inhibitors impaired CB1-mediated bone marrow-derived monocyte/macrophage (BMM) polarization toward M1. The protein expression levels of M1 markers were measured by CBA **(A–C)** and western blot **(D)**. *P < 0.05 compared with control, ^#^*P* < 0.05 compared with the same treated group without inhibitors (*n* = 4).

As a G protein-coupled receptor, the biological function of CB1 depends on multiple signaling pathways, such as mitogen-activated protein kinases (MAPKs) and AMPK signaling pathways (31). Our preceding study noted that CB1 activated RhoA to promote migration and phagocytosis in macrophages (22, 32). To identify the responsible signaling pathways for M1 polarization of BMMs regulated by CB1, we applied pharmacological inhibitors of signal transduction, PTX (G(α)_i/o_ inhibitor), Y27632 (ROCK inhibitor), SB203580 (p38 inhibitor), PD98059 (ERK inhibitor), and compound C (AMPK inhibitor). The CB1-mediated increase of CD86 mRNA expression was apparently impaired by PTX, Y27632, and PD98059 in BMMs, while SB203580 and compound C had no such effect (Figure 7A). Apart from this, PTX, Y27632, and PD98059 also markedly attenuated the mRNA levels of M1 other gene signatures increased by ACEA (Figures 7B–D). PTX, Y27632, and PD98059 pretreatment also decreased the protein levels of MIP-1β, IL-6, TNF (by CBA) (Figures 8A–C), and iNOS (by western blot) (Figure 8D) induced by ACEA. These findings implied that G(α)_i/o_, RhoA, and ERK were involved in macrophage polarization mediated by CB1.

**Figure 9:**
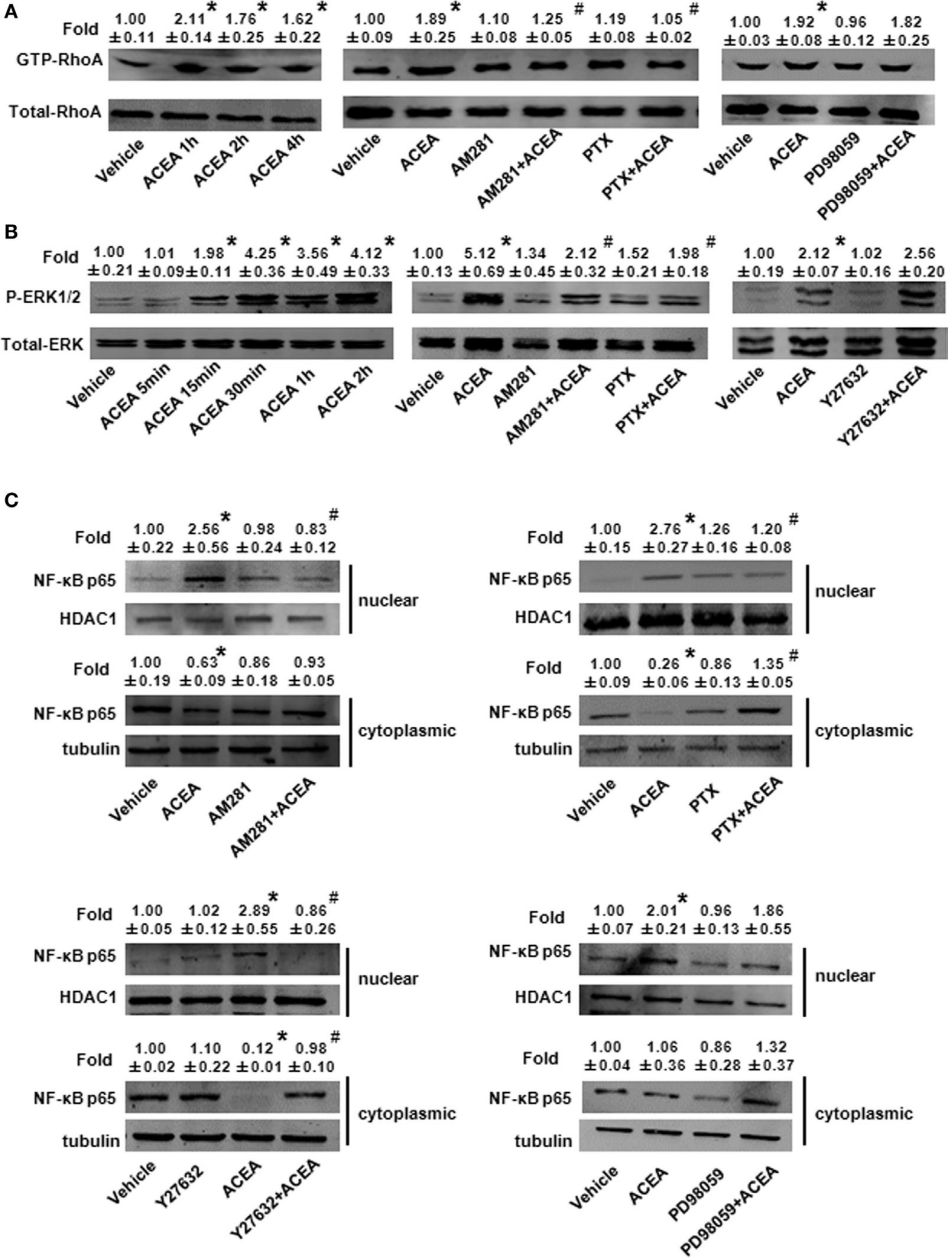
ACEA activated G(α)_i/o_-coupled CB1 and then enlarged GTP-bound Rho and extracellular signal-regulated kinase (ERK) signal, finally promoting bone marrow-derived monocyte/macrophage (BMM) polarization toward M1. *In vitro* total and active Rho proteins **(A)**, total ERK1/2 and phosphor-ERK1/2 **(B)** expression and NF-kB p65 nuclear translocation **(C)** was measured by western blot. *P < 0.05 compared with control, ^#^*P* < 0.05 compared with the same treated group without inhibitors (*n* = 5).

We investigated the effect of CB1 on signal pathway activation of its downstream. pull-down analysis showed that ACEA promoted active GTP-bound Rho protein levels of BMMs in 1, 2, and 4 h and this added GTP-bound RhoA conformation was impaired by AM281 pretreatment. We noted that PTX weakened GTP-bound RhoA protein with ACEA. However, ERK antagonist made no difference on the active GTP-bound Rho protein expression induced by ACEA, which implied that ERK was not involved the effect of CB1 on RhoA activation (Figure 9A).

Meanwhile, we measured the effect of CB1 on ERK activation. ACEA induced a significant increase in the protein level of phosphor-ERK1/2 and kept longer excitation of ERK1/2. Pretreatment with AM281 or PTX inhibited the increase of phosphor-ERK1/2, while Y27632 did not affect the activation of ERK in BMMs, which implied that ERK and RhoA had no influence on their activation of each other (Figure 9B). Taken together, these findings indicated that ACEA-activated G(α)_i/o_ coupled CB1, and then enlarged GTP-bound Rho and phosphor-ERK1/2 independently, finally promoting BMM polarization toward M1 phenotype.

**Figure 10:**
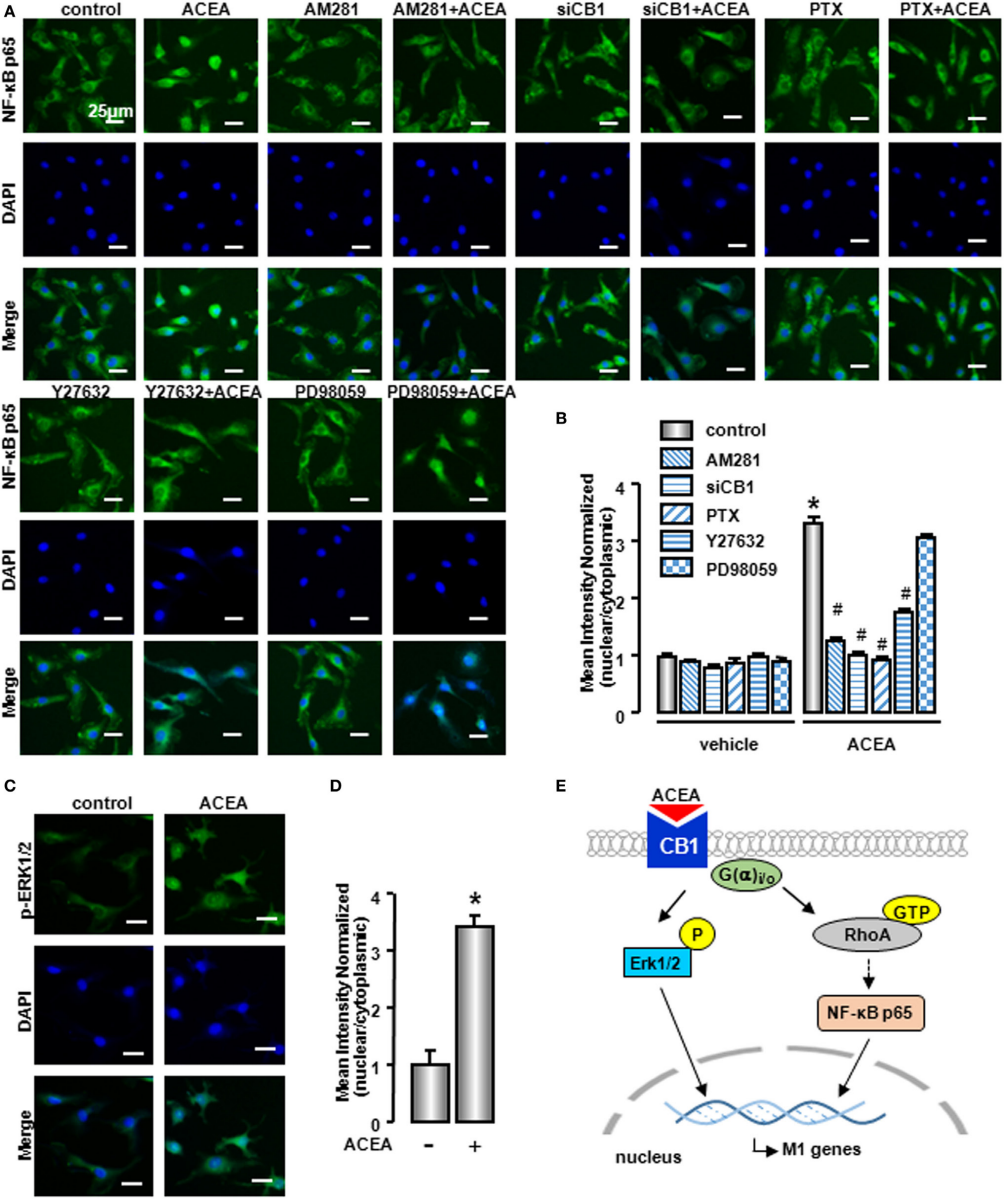
NF-κB activation was involved in M1 polarization mediated by CB1. NF-kB p65 **(A)** and ERK1/2 **(C)** nuclear translocation were evaluated by immunofluorescence. DAPI was used to visualize nuclei (blue). Scale bars, 25 µm. The mean optical density was measured by high content analysis **(B,D)**. *P < 0.05 compared with control, ^#^*P* < 0.05 compared with the same treated group without inhibitors (*n* = 4). Scheme of CB1 promoting bone marrow-derived monocyte/macrophage (BMM) polarization toward M1 phenotype **(E)**.

Owing to the center role of NF-κB p65 nuclear translocation in M1 polarization, we wondered whether it was involved in CB1 regulation on M1 polarization. We detected NF-κB p65 localization in the presence of CB1 agonist by western blot and high content analysis. As shown in Figure 9C and Figure S1D in Supplementary Material, nuclear NF-κB p65 protein was increased in ACEA-treated cells, while cytoplasmic NF-kB p65 was decreased slightly. If the ratio of nuclear to cytoplasmic NF-κB p65 (Nuc/Cyto) in untreated cells was set as 1.00, the Nuc/Cyto was 3.32 in ACEA-treated cells. Furthermore, AM281 markedly attenuated CB1-mediated NF-κB p65 nuclear translocation, as the results of western blot analysis revealed that ACEA-induced NF-κB p65 nuclear translocation was reduced after AM281 pretreatment (Nuc/Cyto = 1.26). Moreover, PTX also weakened the NF-κB p65 nuclear translocation with ACEA (Nuc/Cyto = 0.92), which implied ACEA activated NF-κB p65 depending on G(α)_i/o_. Next, we examined whether phosphor-ERK1/2 and GTP-bound Rho protein was involved in ACEA-induced NF-κB activation. The ACEA-induced nuclear translocation of NF-kB p65 was apparently impaired by Y27632 in BMMs (Nuc/Cyto = 1.78), while PD98059 has no such effect (Nuc/Cyto = 3.11). High content analysis showed similar results (Figures 10A,B). These data demonstrated that CB1-mediated NF-kB p65 nuclear translocation only depending on G(α)_i/o_/RhoA signaling pathway.

To clarify the role of CB1 on ERK activation, we also detected phosphor-ERK1/2 nuclear translocation by high content analysis. Nuclear phosphor-ERK1/2 protein was increased in ACEA-treated macrophages, which indicated that phosphor-ERK1/2 could regulate M1 gene signature expressions directly (Figures 10C,D). In conclusion, CB1 mediated M1 polarization of BMMs, depending on two independent signaling pathways: G(α)_i/o_/RhoA/NF-κB p65 and G(α)_i/o_/ERK1/2 pathways (Figure 10E).

## Discussion

An increasing body of evidence shows that endocannabinoid system is involved in liver fibrogenesis. Our preceding study has demonstrated that blockade of CB1 reduces inflammation and fibrosis of injured liver by suppressing BMM infiltration and activation. In this study, we investigated the effects of CB1 on BMM further, focusing on BMM polarization. We found after CCl_4_ administration, M1 gene signature expressions were significantly elevated, while M2-type markers had little changes. We discovered that CB1 showed a positive correlation with M1 markers. Furthermore, the blockade of CB1 reduced the proportion of M1 type BMMs with no influence on the amount of M1 type of Kupffer cells. Focusing on BMM, we noted that CB1 activation exerted a powerful effect on BMM polarization toward M1 phenotype, which depended on G(α)_i/o_/RhoA and G(α)_i/o_/ERK1/2 signaling pathways, respectively.

M1- and M2-type macrophages play different roles in development of many diseases. Owing to its cytokine secretion ability, M1-polaried macrophages have an important effect on the initial phase of diseases. In contrast, M2 macrophages are involved in parasite containment, promote tissue remodeling and tumor progression, and have immunoregulatory functions. It has been reported M2-type macrophages can promote the development of fibrosis (33). However, in our study, in liver injured patients, we found that M1-type cell was increased. By detecting the mRNA levels of M1 and M2 gene signatures, we noted that M1 gene signature expressions were still at high levels in CCl_4_-induced mouse injured livers for 4 weeks, while that of M2 macrophage maintained at low levels during the injury even in the fibrosis stage. The results indicated that M1-type macrophages have a greater significance on liver fibrogenesis.

The macrophages recruited from BM can produce excessive inflammatory cytokines, induce damaged effects to remnant normal liver tissue and exacerbate tissue injury, which ultimately lead to liver fibrosis or sclerosis. However, the type of BMMs in injured tissues is still undefined. In our study, we found that most of M1 macrophages were derived from BMM and M1 type of BMM proportion was increased significantly, which indicated that M1 type of BMMs probably had an important function in damaged livers.

However, it has been reported that Kupffer cells also participated in liver injury (34). They have high phagocytic ability, allowing them to clear up viruses and bacteria, as well as apoptotic cells and cellular debris (35). They can also secrete some cytokines that can attract and stimulate non-parenchymal cells in the liver, including monocytes, neutrophils, natural killer T cells for immune responses (28, 36). It has been reported that depletion of Kupffer cells prevented the development of diet-induced steatosis and hepatic insulin resistance, both in rats and mice (37). Our results also showed that after CCl_4_ administration, the proportion of M1-polaried Kupffer cells was upregulated slightly. Furthermore, some research found that Kupffer cells have an effect on tissue repair stage, which may imply that M2-type macrophages are the majority kind of Kupffer cells (38). Moreover, our preceding study has shown that blockade of CB1 *in vivo* had no effect on the amount of Kupffer cells (22). In this study, we also noticed that CB1 antagonist pretreatment had no influence on Kupffer cell polarization toward M1 phenotype *in vivo*. We also performed CLs injection to delete Kupffer cells. Blockade of CB1 on reducing M1 marker expressions were not affected after Kupffer cell deletion. But, the blockade of CB1 has strong effects on the migration, cytokine secretion, and polarization of BMMs, which provides a novel evidence of macrophage heterogeneity. Further research is needed to discover the role of CB1 on Kupffer cells function *in vivo* and *in vitro*.

It has been reported that CB2, which is predominantly expressed in immune cells, can regulate Kupffer cell polarization toward M2 type. CB2 activation reduces IL-17-induced M1 polarization in macrophages (39). Although CB1 is weakly expressed in immune cells, it can also regulate macrophage functions. During the progression of diabetes, CB1 promotes the Nlrp3-ASC inflammasome activation and the release of IL-1β and IL-18 in infiltrating macrophages, which act as paracrine signals to induce beta cell apoptosis (5). Furthermore, our previous study has reported that CB1 but not CB2 was involved in the migration and activation of BMMs. The blockade of CB1 suppressed BMM infiltration (22). In addition, we also discovered that CB1 mediated the macrophage phagocytosis (32). In this study, we showed that CB1 activation promoted M1 type polarization of BMMs.

A network of signaling molecules, transcription factors, epigenetic mechanisms, and posttranscriptional regulators underlies the polarization of macrophages. IFN and toll-like receptor-activated IRF-STAT1-signaling pathways are involved in regulating macrophages toward the M1 phenotype. However, IL-4 and IL-13-STAT6 signaling pathways orient macrophage function toward M2 phenotype (40). A predominance of NF-κB activation promotes M1 macrophage polarization, resulting in M1 gene signature expressions. NF-κB p65 are involved in the production of pro-inflammatory cytokines (41). A predominance of NF-κB and STAT1 activation promotes M1 macrophage polarization, resulting in cytotoxic and inflammatory functions (3). It has been reported that IL-6 mediated macrophage polarization through STAT5- NF-κB p65 signaling pathway (42). Therefore, we also analyzed the mechanism of M1 polarization reduced by ACEA and realized NF-κB p65 nuclear translocation was involved in this process. But, we need to do some research to figure out the role of CB1 on NF-κB signaling pathway in this process. Furthermore, we found that Rho-GTP influenced the NF-κB p65 nuclear translocation. However, whether Rho-GTP directly influenced NF-κB signaling pathway needs us to do some more experiments in the future.

CB1, belonging to a member of G protein-coupled receptors, is characterized by seven transmembrane helices. It has been found that the major mediators of CB1 belong to G(α) family (31). In our study, PTX (G(α)_i/o_ inhibitor) pretreatment attenuated the mRNA and proteins levels of M1 gene signatures induced by ACEA in BMMs, which implied that G(α)_i/o_ was involved in CB1-mediated BMM polarization toward M1 phenotype. Upon cannabinoids binding to CB1 can activate many signaling pathways to exert its effect. In this study, we discovered the CB1-mediated increase of CD86 mRNA expression was apparently impaired by RhoA-ROCK inhibitor and ERK inhibitor in BMMs. However, p38 inhibitor and AMPK inhibitor had no such effect. But p38 and AMPK signaling pathways are involved in other CB1 functions. For instance, activation of CB1 by endocannabinoids may play an important role in the pathogenesis of diabetic cardiomyopathy by facilitating p38-MAPK activation (18). And CB1 could regulate neuroendocrine differentiation of prostate cancer depending on AMPK signaling pathway (43). Therefore, CB1 exerts different functions in various kinds of cell and tissues, depending on different signaling pathways.

In summary, our data showed that CB1 activation promoted M1 polarization of BMMs, depending on G(α)_i/o_/RhoA and G(α)_i/o_/ERK1/2 signaling pathways, respectively. CB1 activation could also promote NF-κB p65 nuclear translocation. The blockade of CB1 reduced the quantity of M1 type of BMMs. This study demonstrates a novel biological mechanism of treating liver fibrogenesis by aiming at CB1 of BMMs.

## Ethics Statement

All animal work was conformed to the Ethics Committee of Capital Medical University and in accordance with the approved guidelines (approval number: AEEI-2014-131).

## Author Contributions

LT: acquisition of data; analysis and interpretation of data; drafting of the manuscript; WL, Le Y, NC, XF, XJ, JX, Lin Y: acquisition of data; material support; LL: study concept and design, financial support; drafting of the manuscript.

## Conflict of Interest Statement

The authors declare that the research was conducted in the absence of any commercial or financial relationships that could be construed as a potential conflict of interest.

## Abbreviations

CBs, cannabinoid receptors; BM, bone marrow; BMM, BM-derived monocyte/macrophage; CCl_4_, carbon tetrachloride; CBA, cytometric bead array; ERK, extracellular signal-regulated kinase; IFN-γ, interferon-γ; MIP-1β, macrophage inflammatory protein-1β; IL, interleukin; iNOS, inducible nitric oxide synthase; TNF, tumor necrosis factor; Arg1, arginase1; 2-AG, 2-arachidonoylglycerol; MAPK, mitogen-activated protein kinase; AMPK, adenosine monophosphate-activated protein kinase; TLRs, toll-like receptors; PTX, pertussis toxin; FACS, fluorescence-activated cell sorting; CL, clodronate liposome.

## References

[1] Tacke F Zimmermann HW. Macrophage heterogeneity in liver injury and fibrosis. J Hepatol (2014) 60:1090–6. 10.1016/j.jhep.2013.12.025 24412603

[2] Sica A Invernizzi P Mantovani A. Macrophage plasticity and polarization in liver homeostasis and pathology. Hepatology (2014) 59:2034–42. 10.1002/hep.26754 24115204

[3] Sica A Mantovani A. Macrophage plasticity and polarization: in vivo veritas. J Clin Invest (2012) 122:787–95. 10.1172/JCI59643 22378047 PMC3287223

[4] Biswas SK Chittezhath M Shalova IN Lim JY. Macrophage polarization and plasticity in health and disease. Immunol Res (2012) 53:11–24. 10.1007/s12026-012-8291-9 22418728

[5] Jourdan T Godlewski G Cinar R Bertola A Szanda G Liu J Activation of the Nlrp3 inflammasome in infiltrating macrophages by endocannabinoids mediates beta cell loss in type 2 diabetes. Nat Med (2013) 19:1132–40. 10.1038/nm.3265 23955712 PMC4050982

[6] Zhuge F Ni Y Nagashimada M Nagata N Xu L Mukaida N DPP-4 inhibition by linagliptin attenuates obesity-related inflammation and insulin resistance by regulating M1/M2 macrophage polarization. Diabetes (2016) 65(10):2966–79. 10.2337/db16-0317 27445264

[7] Varin A Gordon S Alternative activation of macrophages: immune function and cellular biology. Immunobiology (2009) 214:630–41. 10.1016/j.imbio.2008.11.009 19264378

[8] Martinez FO Helming L Gordon S. Alternative activation of macrophages: an immunologic functional perspective. Annu Rev Immunol (2009) 27:451–83. 10.1146/annurev.immunol.021908.132532 19105661

[9] Porta C Riboldi E Ippolito A Sica A. Molecular and epigenetic basis of macrophage polarized activation. Semin Immunol (2015) 27:237–48. 10.1016/j.smim.2015.10.003 26561250

[10] Mantovani A Biswas SK Galdiero MR Sica A Locati M Macrophage plasticity and polarization in tissue repair and remodelling. J Pathol (2013) 229:176–85. 10.1002/path.4133 23096265

[11] Fonseca BM Costa MA Almada M Correia-da-Silva G Teixeira NA Endogenous cannabinoids revisited: a biochemistry perspective. Prostaglandins Other Lipid Mediat (2013) 10(2–103):13–30. 10.1016/j.prostaglandins.2013.02.002 23474290

[12] Howlett AC Breivogel CS Childers SR Deadwyler SA Hampson RE Porrino LJ. Cannabinoid physiology and pharmacology: 30 years of progress. Neuropharmacology (2004) 47(Suppl 1):345–58. 10.1016/j.neuropharm.2004.07.030 15464149

[13] Cinar R Godlewski G Liu J Tam J Jourdan T Mukhopadhyay B Hepatic cannabinoid-1 receptors mediate diet-induced insulin resistance by increasing de novo synthesis of long-chain ceramides. Hepatology (2014) 59:143–53. 10.1002/hep.26606 23832510 PMC3839256

[14] Lecru L Desterke C Grassin-Delyle S Chatziantoniou C Vandermeersch S Devocelle A Cannabinoid receptor 1 is a major mediator of renal fibrosis. Kidney Int (2015) 88:72–84. 10.1038/ki.2015.63 25760323

[15] Liu LY Alexa K Cortes M Schatzman-Bone S Kim AJ Mukhopadhyay B Cannabinoid receptor signaling regulates liver development and metabolism. Development (2016) 143:609–22. 10.1242/dev.121731 26884397 PMC4760316

[16] Mazier W Saucisse N Gatta-Cherifi B Cota D The endocannabinoid system: pivotal orchestrator of obesity and metabolic disease. Trends Endocrinol Metab (2015) 26:524–37. 10.1016/j.tem.2015.07.007 26412154

[17] Mukhopadhyay B Schuebel K Mukhopadhyay P Cinar R Godlewski G Xiong K Cannabinoid receptor 1 promotes hepatocellular carcinoma initiation and progression through multiple mechanisms. Hepatology (2015) 61:1615–26. 10.1002/hep.27686 25580584 PMC4406817

[18] Rajesh M Batkai S Kechrid M Mukhopadhyay P Lee WS Horvath B Cannabinoid 1 receptor promotes cardiac dysfunction, oxidative stress, inflammation, and fibrosis in diabetic cardiomyopathy. Diabetes (2012) 61:716–27. 10.2337/db11-0477 22315315 PMC3282820

[19] Suk KT Mederacke I Gwak GY Cho SW Adeyemi A Friedman R Opposite roles of cannabinoid receptors 1 and 2 in hepatocarcinogenesis. Gut (2016) 65:1721–32. 10.1136/gutjnl-2015-310212 27196571 PMC6594387

[20] Martella A Silvestri C Maradonna F Gioacchini G Allara M Radaelli G Bisphenol A induces fatty liver by an endocannabinoid-mediated positive feedback loop. Endocrinology (2016) 157:1751–63. 10.1210/en.2015-1384 27014939 PMC6285285

[21] Tam J Liu J Mukhopadhyay B Cinar R Godlewski G Kunos G. Endocannabinoids in liver disease. Hepatology (2011) 53:346–55. 10.1002/hep.24077 21254182 PMC3073545

[22] Mai P Yang L Tian L Wang L Jia S Zhang Y Endocannabinoid system contributes to liver injury and inflammation by activation of bone marrow-derived monocytes/macrophages in a CB1-dependent manner. J Immunol (2015) 195:3390–401. 10.4049/jimmunol.1403205 26320250

[23] Bambang KN Lambert DG Lam PM Quenby S Maccarrone M Konje JC. Immunity and early pregnancy events: are endocannabinoids the missing link? J Reprod Immunol (2012) 96:8–18. 10.1016/j.jri.2012.10.003 23177537

[24] Eisenstein TK Meissler JJ. Effects of cannabinoids on T-cell function and resistance to infection. J Neuroimmune Pharmacol (2015) 10:204–16. 10.1007/s11481-015-9603-3 25876735 PMC4470840

[25] Duncan M Galic MA Wang A Chambers AP McCafferty DM McKay DM Cannabinoid 1 receptors are critical for the innate immune response to TLR4 stimulation. Am J Physiol Regul Integr Comp Physiol (2013) 305:R224–31. 10.1152/ajpregu.00104.2013 23739343

[26] Jourdan T Godlewski G Kunos G Endocannabinoid regulation of beta-cell functions: implications for glycaemic control and diabetes. Diabetes Obes Metab (2016) 18:549–57. 10.1111/dom.12646 26880114 PMC5045244

[27] Katchan V David P Shoenfeld Y. Cannabinoids and autoimmune diseases: a systematic review. Autoimmun Rev (2016) 15:513–28. 10.1016/j.autrev.2016.02.008 26876387

[28] Heymann F Tacke F Immunology in the liver – from homeostasis to disease. Nat Rev Gastroenterol Hepatol (2016) 13:88–110. 10.1038/nrgastro.2015.200 26758786

[29] Wuestefeld T Pesic M Rudalska R Dauch D Longerich T Kang TW A direct in vivo RNAi screen identifies MKK4 as a key regulator of liver regeneration. Cell (2013) 153:389–401. 10.1016/j.cell.2013.03.026 23582328

[30] Park JK Shao M Kim MY Baik SK Cho MY Utsumi T An endoplasmic reticulum protein, Nogo-B, facilitates alcoholic liver disease through regulation of Kupffer cell polarization. Hepatology (2017) 65:1720–34. 10.1002/hep.29051 28090670 PMC5397326

[31] Turu G Hunyady L. Signal transduction of the CB1 cannabinoid receptor. J Mol Endocrinol (2010) 44:75–85. 10.1677/JME-08-0190 19620237

[32] Mai P Tian L Yang L Wang L Yang L Li L Cannabinoid receptor 1 but not 2 mediates macrophage phagocytosis by G(alpha)i/o/RhoA/ROCK signaling pathway. J Cell Physiol (2015) 230:1640–50. 10.1002/jcp.24911 25545473

[33] Braga TT Agudelo JS Camara NO. Macrophages during the fibrotic process: M2 as friend and foe. Front Immunol (2015) 6:602. 10.3389/fimmu.2015.00602 26635814 PMC4658431

[34] Sato K Hall C Glaser S Francis H Meng F Alpini G Pathogenesis of Kupffer cells in cholestatic liver injury. Am J Pathol (2016) 186:2238–47. 10.1016/j.ajpath.2016.06.003 27452297 PMC5012503

[35] Gao B Jeong WI Tian Z. Liver: an organ with predominant innate immunity. Hepatology (2008) 47:729–36. 10.1002/hep.22034 18167066

[36] Gregory SH Cousens LP van Rooijen N Dopp EA Carlos TM Wing EJ. Complementary adhesion molecules promote neutrophil-Kupffer cell interaction and the elimination of bacteria taken up by the liver. J Immunol (2002) 168:308–15. 10.4049/jimmunol.168.1.308 11751975

[37] Ni Y Zhuge F Nagashimada M Ota T Novel action of carotenoids on non-alcoholic fatty liver disease: macrophage polarization and liver homeostasis. Nutrients (2016) 8:E391 10.3390/nu8070391 27347998 PMC4963867

[38] Bleriot C Dupuis T Jouvion G Eberl G Disson O Lecuit M. Liver-resident macrophage necroptosis orchestrates type 1 microbicidal inflammation and type-2-mediated tissue repair during bacterial infection. Immunity (2015) 42:145–58. 10.1016/j.immuni.2014.12.020 25577440

[39] Louvet A Teixeira-Clerc F Chobert MN Deveaux V Pavoine C Zimmer A Cannabinoid CB2 receptors protect against alcoholic liver disease by regulating Kupffer cell polarization in mice. Hepatology (2011) 54:1217–26. 10.1002/hep.24524 21735467

[40] Wang N Liang H Zen K. Molecular mechanisms that influence the macrophage m1-m2 polarization balance. Front Immunol (2014) 5:614. 10.3389/fimmu.2014.00614 25506346 PMC4246889

[41] Cao X. Self-regulation and cross-regulation of pattern-recognition receptor signalling in health and disease. Nat Rev Immunol (2016) 16:35–50. 10.1038/nri.2015.8 26711677

[42] Kawashima T Murata K Akira S Tonozuka Y Minoshima Y Feng S STAT5 induces macrophage differentiation of M1 leukemia cells through activation of IL-6 production mediated by NF-kappaB p65. J Immunol (2001) 167:3652–60. 10.4049/jimmunol.167.7.3652 11564778

[43] Morell C Bort A Vara D Ramos-Torres A Rodriguez-Henche N Diaz-Laviada I. The cannabinoid WIN 55,212-2 prevents neuroendocrine differentiation of LNCaP prostate cancer cells. Prostate Cancer Prostatic Dis (2016) 19:248–57. 10.1038/pcan.2016.19 27324222 PMC5411672

