# Supplementary figures and images for: Cannabinoid Receptor 1 Participates in Liver Inflammation by Promoting M1 Macrophage Polarization via RhoA/NF-κB p65 and ERK1/2 Pathways, Respectively, in Mouse Liver Fibrogenesis
Source: Front Immunol. 2017 Sep 28;8:1214. doi: 10.3389/fimmu.2017.01214 (PMC5625548; doi:10.3389/fimmu.2017.01214)

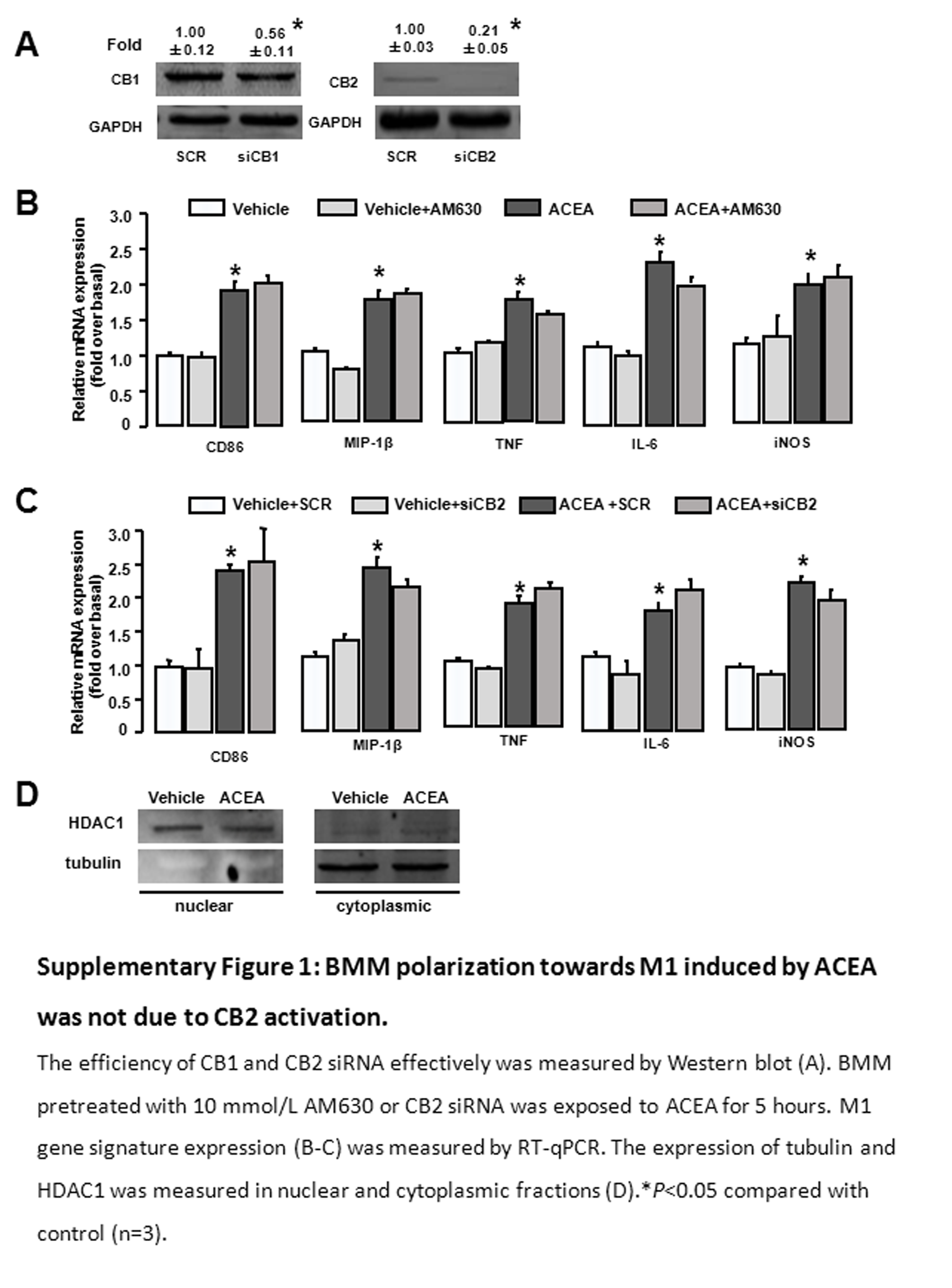

Supplement: Supplementary file 1 [file image_1.tif]
